# Simple deep sequencing-based post-remission MRD surveillance predicts clinical relapse in B-ALL

**DOI:** 10.1186/s13045-018-0652-y

**Published:** 2018-08-22

**Authors:** Shuhua Cheng, Giorgio Inghirami, Shuo Cheng, Wayne Tam

**Affiliations:** 1 Department of Pathology and Laboratory Medicine, Weill Cornell Medicine, New York, NY 10021 USA; 2 Department of Computer Science, School of Engineering, Cornell University, Ithaca, New York, NY 14853 USA

**Keywords:** NGS, MRD surveillance, B lymphoblastic leukemia/lymphoma, Relapse, Post-remission setting

## Abstract

**Background:**

Next-generation sequencing (NGS) of the rearranged immunoglobulin heavy-chain gene has emerged as a highly sensitive method to detect minimal residual disease (MRD) in B acute lymphoblastic leukemia/lymphoma (B-ALL). However, a sensitive and easily implemented NGS methodology for routine clinical laboratories is lacking and clinical utility of NGS-MRD surveillance in a post-remission setting to predict clinical relapse has not been determined.

**Methods:**

Here we described a simple and quantitative NGS platform and assessed its performance characteristics, quantified NGS-MRD levels in 122 B-ALL samples from 30 B-ALL patients, and explored the clinical merit of NGS-based MRD surveillance.

**Results:**

The current NGS platform has an analytic sensitivity of 0.0001% with excellent specificity and reproducibility. Overall, it performs better than routine multi-color flow cytometry (MCF) in detecting MRD. Utilizing this assay in MRD surveillance in a post-remission setting showed that it detected conversion to positive MRD (CPMRD) in patients with NGS-based molecular remission much earlier than MCF, and that positive MRD conversion could be detected as early as 25.6 weeks prior to clinical relapse in closely surveilled patients. Post-remission CPMRD, but not NGS-based MRD positivity at end of induction, can accurately predict clinical relapse in our limited cohort of B-ALL patients.

**Conclusions:**

This pilot proof-of-concept study illustrates the clinical utility of a simple, sensitive, and clinically feasible MRD detection platform in post-remission NGS-based MRD surveillance and early relapse detection in B-ALL patients.

**Electronic supplementary material:**

The online version of this article (10.1186/s13045-018-0652-y) contains supplementary material, which is available to authorized users.

## Background

Minimal residual disease has emerged to be an important biomarker for risk stratification and individual risk-directed treatment decision in B acute lymphoblastic leukemia/lymphoma (B-ALL). Studies with large cohort of pediatric B-ALL patients demonstrated that minimal residual disease (MRD) status is a powerful predictor for relapse and clinical outcome [1–4]. In adults with relapsed/refractory B-ALL, MRD negativity after salvage therapy is associated with significantly longer overall survival (OS) [5]. Protocols of high-risk patients designed based on MRD readouts led to a fivefold increase of 5-year event-free survival (EFS) rate without recurrence. Two conventional approaches for detection of MRD in B-ALL have been multi-parameter flow cytometry (MFC) and qPCR, each of which has distinct limitations [6]. MFC is a relatively simple procedure with short turnaround time, and it is currently the most frequently applied modality to quantify MRD in clinical laboratories across the USA. However, technical constraints, for example, sample availability, low tumor burden, immunophenotypic shifts, and clonal selection, can decrease its sensitivity leading to false negative results, as suggested by the higher than expected rate of relapses in negative MFC-MRD [3, 7]. The second conventional method is the allele-specific oligonucleotide PCR (ASO-PCR). This methodology requires the design of customized patient-specific primers in the VDJ junctions of the *IGH* gene and individual optimization of testing conditions to monitor MRD; thus, the procedure is laborious and time-consuming, and not routinely available in the USA. Importantly, some adult patients with flow or qPCR-based MRD negativity at the end of induction or after consolidation treatment relapse clinically and a fraction of the patients with flow or qPCR-based MRD positivity remained in complete remission (CR) without hematologic recurrence [8, 9]. A more reliable, sensitive, and dynamic MRD detection methodology is needed.

Recently, several studies have explored next-generation sequencing (NGS)-based deep sequencing assays for the determination of MRD in B-ALL patients [10–17]. Similar to ASO-PCR, this method utilizes the unique sequences within the VDJ junctions in B-lymphocytes as unique/clonal markers to identify and track MRD [18–20]. However, unlike ASO-PCR, the NGS-based VDJ deep sequencing method interrogates leukemic-specific (*IGH*) VDJ gene rearrangement without a need of customized PCR primers and conditions. Pioneering studies demonstrated excellent sensitivity and reliability of the NGS-MRD detection method [10–17]. Prognostic significance and predictive power of NGS-MRD status during induction therapy or at the end of induction or in bone marrow transplantation setting have been confirmed in B-ALL, particularly for pediatric patients [11, 13, 14, 21]. Besides MRD monitoring after induction, MRD monitoring in a post-remission setting may become increasingly relevant. This is evidenced by recent findings which showed a correlation between favorable clinical outcome and low disease burden in relapsed B-ALL patients treated with preemptive therapy like CAR T immunotherapy [22], which imply that early relapse detection might be beneficial. The clinical relevance and utility of NGS-based MRD surveillance in a post-remission setting has not been previously determined. Though the NGS-based MRD test described in recent studies is commercially available, its accessibility is limited to sample send-out to a central laboratory. The methodology is proprietary, not easily replicable, and cannot be easily adopted in routine clinical molecular pathology laboratories. Thus the development of a highly sensitive, reproducible, quantitative assay that can be readily implemented and adopted for routine MRD surveillance seems warranted.

Here we describe a simple, ultrasensitive, and easily applicable NGS assay with excellent performance. We also explore the clinical utility and merit of this assay in post-remission MRD surveillance to generate biomarkers for early relapse detection.

## Methods

### Sample preparation

A total of 128 cryopreserved clinical samples from 32 B-ALL patients (32 initial diagnostic, relapse, and additional 96 post-treatment specimens) were initially retrieved from the biobank of the Department of Pathology and Laboratory Medicine at Weill Cornell Medicine and evaluated by this study. Clinical Information was obtained from electronic clinical records. This study was conducted in accordance with the Declaration of Helsinki regulations of the protocols approved by the Institutional Review Board of Weill Cornell Medicine, New York, USA. Written consent for use of the samples for research was obtained from patients or their guardians.

### DNA extraction and concentration

Genomic DNA was extracted from bone marrow and PBMC cell pellets following manufacturer’s instructions (QIAamp DNA Mini Kit, Qiagen, Germantown). If necessary, DNA samples were concentrated using a Genomic DNA Clean & Concentrator-10 column (D4010, Zymo Research, Irvine). DNA samples and sequencing libraries were quantitated by Tape Station (Agilent Technologies, Santa Clara) and Qubit (Thermo Fisher Scientific, Singapore).

### MRD detection by conventional flow cytometry

MRD by MFC was assessed using the Euroflow 8-color panel on bone marrow specimens obtained at clinical remission and at approximately 1–6-month intervals. For each of the specimens tested, between 400,000 and 1 million events (excluding debris) were initially acquired. Doublet exclusion was performed by plotting the height against the area for forward scatter and single cells (singlets) were accordingly gated for further analysis.

### MRD detection with LymphoTrack-Miseq platform

Deep sequencing by LymphoTrack® IGHV Leader Somatic Hypermutation Assay-MiSeq/IGH FR1/2/3 Panel-MiSeq (71,210,069/71210139, Invivoscribe) (LIGV-Miseq) was performed following the manufacturer’s instructions with modifications to improve the MRD quantification. The overall methodology of the assay is summarized in Additional file 1. Briefly, a set of primers targeting the Leader (VHL) or FR1/3 and *J*_*H*_ gene regions of *IGH* were contained in a single multiplex master mix in which the designed primers included unique Illumina adaptor index. For diagnostic samples, 0.02–0.5 μg of genomic DNA was used in a 29–31 μl one-step PCR reaction (25 μl Master mix+4 μl genomic DNA, or 25 μl Master mix+4 μl genomic DNA + 1–2 μl MRD control spike-in). For any given follow-up samples, all available amounts of DNA with a range of 0.5 to 5 μg of genomic DNA were used in a 45–47 μl one-step PCR reaction (39 μl Master mix+6 μl genomic DNA, or 39 μl Master mix+6 μl genomic DNA + 2 μl MRD control spike-in). MRD control spike-in contained the equivalent amount of DNA from 50 to 500 B-lymphoid cells with monoclonal *IGH* rearrangement. After PCR reaction, amplified VDJ amplicons were mixed with 1 volume of Agencourt AMPure XP beads (Beckman Coulter) for 5 min at room temperature. Mixed samples were placed on a DynaMag 96-well plate (5 min) and then washed with 200 μl of 80% ethanol twice, following by elution with 20 μl of 10 mM Tris buffer (pH 8.0). The eluted libraries then were mixed with 18 μl of AMPure XP beads again, and the binding and washing procedures were repeated. The second elution was conducted with 15 μl of 10 mM Tris buffer. Quality and quantity of purified VDJ sequencing libraries were assessed with Tape station system (Agilent Technologies) and Qubit (Thermo Fisher Scientific). Pooled libraries (10~15 pM) were loaded into Reagent Cartridge (MiSeq Reagent Kits v3, Illumina) and sequenced (600 cycles) using a Miseq unit (Illumina). Libraries generated from the diagnostic samples are sequenced separately in different runs from those generated from post-treatment samples to avoid bioinformatics contamination due to read mis-assignment.

### Sequencing data analysis

Fastq files were initially analyzed with the LymphoTrack-Miseq software from Invivoscribe following the manufacturer’s guideline. This analysis identifies VDJ sequences from diagnostic samples and creates an output that includes all unique VDJ sequences and their corresponding abundance. The dominant B-ALL tumor clone, as well as any minor subclones that generated 5% or more of total reads, was identified from these sequences. We developed a custom algorithm that used leukemia-specific VDJ junction sequences, defined as the complementarity-determining region 3 (CDR3) of the dominant B-cell tumor clone and subclones (if present) to identify MRD in post-treatment samples with ultra-high sensitivity. The algorithm does not tolerate any mismatch in the junction sequences for MRD detection. Although theoretically the entire VDJ sequence can also be used for MRD tracking, we found that the use of VDJ junction sequence had superior sensitivity. If more than one clone is identified in a diagnostic sample, the same algorithm run will be repeated for each of the independent leukemia-specific VDJ junctions in any follow-up samples. Matched reads of more than two were considered positive. Tumor load was calculated based on one of the two methods: (1) MRD% = (number of leukemia cell-specific VDJ reads/total numbers of VDJ read mapped in a sample) × (corresponding fraction of B cells defined as CD19 + % in the total mononuclear cell population as determined by flow cytometry) × 100; (2) MRD% = (number of leukemia cell-specific VDJ reads/number of VDJ reads generated from MRD control spike-in) × (number of cells corresponding to MRD control spike-in input (50–500))/total number of cells tested in a given sample × 100. The current assay along with the LymphoTrack-Miseq software might not completely exclude potential multiplex PCR amplification bias (i.e., over- or under-representation of certain VDJ recombination) due to unknown and proprietary primer sequences and analysis strategy Invivoscribe applied.

### Definition and assessment of negative and positive MRD conversion status

To investigate the clinical predictive value of post remission MRD surveillance in B-ALL patients, MRD trends during the post remission period was divided into two categories: conversion to positive MRD (CPMRD), and negative for MRD conversion (NMRDC). CPMRD was defined when the NGS-MRD became detectable any time post-treatment after initially achieving negative MRD by NGS, and NMRDC was defined when patient’s NGS-MRD levels reached undetectable levels and remained as such in the post-treatment period up to Jan. 20, 2018. Categorization to NMRDC or CPMRD was only possible when two or more sequential clinical follow-up samples for each of the B-ALL patients were available.

### Statistical methods

Linear regression and Pearson correlation were used to analyze the sensitivity of the NGS test and to compare tumor burden measurements obtained by multi-color flow cytometry (MCF) and NGS. Relapse-free survival comparison between patient groups (CPMRD vs NMRDC) was performed using Kaplan-Meier curves (log-rank test, significance defined as *p* < 0.05). Significant differences between categorical variables, clinical specificity, clinical sensitivity, positive predictive value (PPV), and negative predictive value (NPV) were calculated with contingency 2 × 2 table. Survival graphs and linear plots were generated using GraphPad/Prism 5 software.

## Results

### Performance characteristic of LIGV-Miseq MRD detection method

**Fig. 1:**
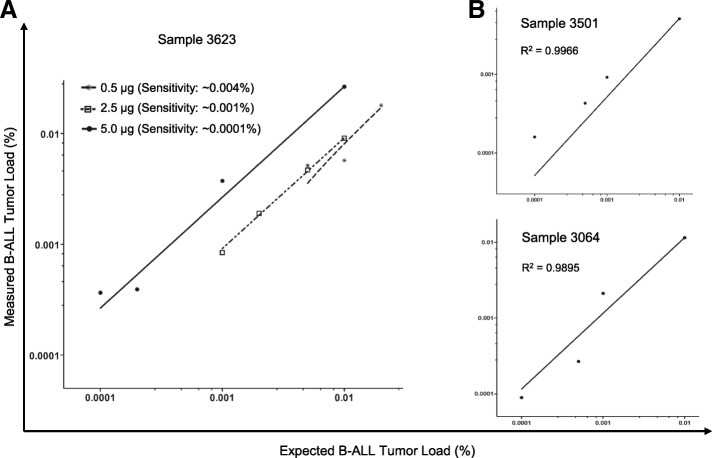
Determination of optimal DNA input and analytical sensitivity. **a**. Effect of DNA input amount on assay sensitivity. **b** Linearity and sensitivity of the assay. Tenfold serial dilution of B-ALL diagnostic DNA samples (**a** 3623; **b** 3501 and 3064) with peripheral blood DNA from healthy individuals was performed, followed by deep VDJ sequencing procedure using a total DNA input of indicated amounts (**a**) or 5 μg (**b**) per sample. With 5 μg DNA input, the sensitivity of the assay can reach 10^− 6^

To assess the sensitivity of our NGS-MRD assay and to define an optimal amount of input DNA, serially diluted clinical samples containing a broad range of leukemic cells from 0.1 to 0.00005% were subjected to sequencing library preparation with different amounts of DNA input (0.5 μg, 2.5 μg, and 5μg) and MRD level quantification in duplicate or triplicate. As shown in Fig. 1a, the sensitivity of the assay was enhanced with increased DNA input. Specifically, inputs of 0.5 μg, 2.5 μg, and 5 μg resulted in analytical sensitivities of ~ 0.004%, ~ 0.001%, and ~ 0.0001%, respectively, corresponding to the capability of the assay to detect 50, 10, and 1 leukemic cells among 1 million normal leukocytes. Therefore, ~ 5 μg DNA is recommended as the input amount for routine clinical testing to maximize sensitivity if enough DNA material is available. With this DNA input, the assay reproducibly detected in all three samples the leukemic clone-specific *IGH* rearrangements with a sensitivity of 2 × 10^− 6^, and in at least one of the replica at the 1 × 10^− 6^ dilution (Fig. 1b and Additional file 2). Overall, the dilution test showed an excellent linearity and high correlation between expected and observed clonal frequencies (*r*^2^ > 0.98), as well as superior sensitivity (~ 0.0001%) in the presence of adequate DNA input.

**Fig. 2:**
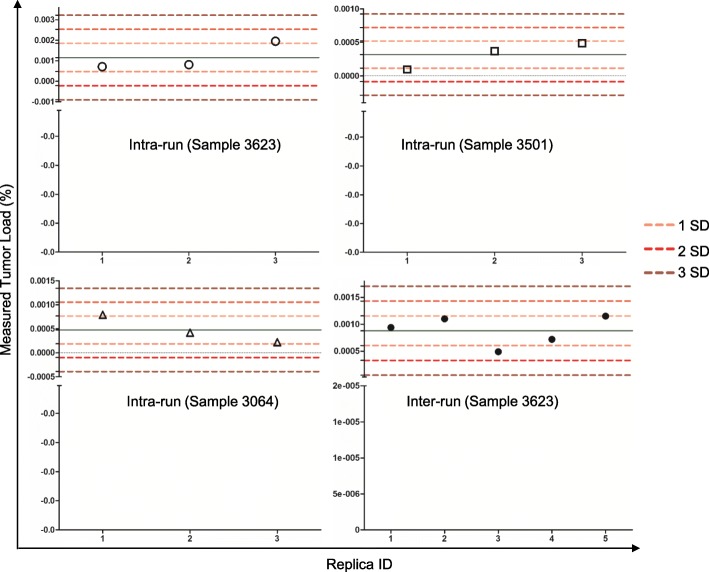
Assessment of intra-run and inter-run reproducibility. Three samples (3623, 3064, and 3501) were run in triplicates to measure intra-run reproducibility. Sample 3623 was assayed in five separate runs to measure inter-run reproducibility. SD, standard deviation. All values obtained are within 2 SD of the mean

To investigate the intra-run reproducibility of the assay, the same B-ALL samples diluted to 2 × 10^− 6^, which is near the limit of detection of the assay, and MRD levels were measured in triplicate in a single run. Our results showed that the intra-run variation was relatively small (Fig. 2 and Additional file 3). Specifically, for samples 3623, 3501, and 3064, the mean ± SD of the MRD levels were 0.00116% ± 0.000689%, 0.00032 ± 0.00020%, and 0.00048 ± 0.000289%, respectively. All the MRD values measured were within 2SD from mean with a median coverage of 432,556×. To evaluate the inter-run reproducibility, a B-ALL sample (3623) with a MRD level of ~ 0.001% was repeatedly measured in five independent runs on five separate days. The mean value for the five separate runs is 0.00088% ± 0.000275% (mean ± SD) with a median coverage of 129,000× (Fig. 2 and Additional file 4). These data support a high precision of the assay in both intra-run and inter-run settings.

**Fig. 3:**
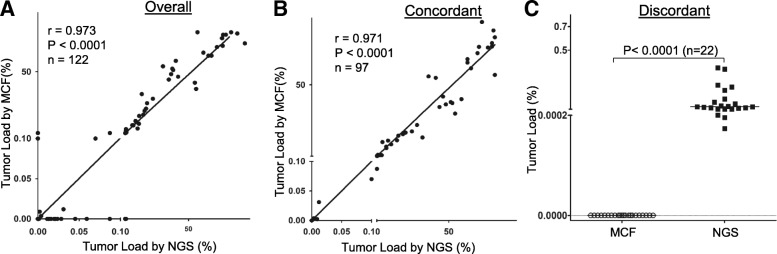
Comparison of tumor load determined by the NGS and MCF assays. Correlation between NGS and MCF-based measurement of tumor load is illustrated for all samples (**a**), the concordant samples (**b**), and the MCF-negative/NGS-positive discordant samples (**c**). r, correlation coefficient, n, number of samples, p, *p* value

The diagnostic accuracy of the assay was evaluated by performing comparative studies between the NGS and conventional eight-color MCF assays. For this purpose, a total number of 128 B-ALL samples from 32 B-ALL patients, including diagnostic (Dx, *n* = 22), 10 relapse, and 96 post-treatment follow-up specimens, were selected. Among these 128 samples, 6 samples from 2 patients were excluded because clonal *IGH* sequences could not be identified for their diagnostic samples. The remaining 122 specimens (Dx, 20, follow-up, 92, relapse, 10) from 30 patients (93.8% of the patients tested, see Additional file 5 for patient characteristics) were analyzed by both eight-color MFC and NGS for tumor content (%). Two independent methods were available to calculate MRD levels (flow cytometry vs spike-in) depending on whether the flow cytometry data for B cell fraction in a given sample was available. A comparison study with 20 B-ALL samples from 7 patients showed that these two calculation methods produced comparable results with excellent correlation and linearity (*r* = 0.99, *p* < 0.0001) (Additional file 6).Overall, there was excellent concordance between the NGS and MFC assays for tumor burden levels (Fig. 3a and Additional file 7). Of the 122 evaluable samples from 30 patients with a median coverage of 395,542 reads per sample, 98 (80.3%), consisting of 45 (36.9%) positive and 53 (43.4%) negative samples, were qualitatively (positive vs negative) and quantitatively concordant. Correlation of the measured tumor burdens between the two methods in the entire cohort as well as in the concordant cases was very high (*p* < 0.0001, *r* = 0.971 and 0.973, respectively) (Fig. 3a, b). This rate of concordance is consistent with previously reported results [17, 23].

**Fig. 4:**
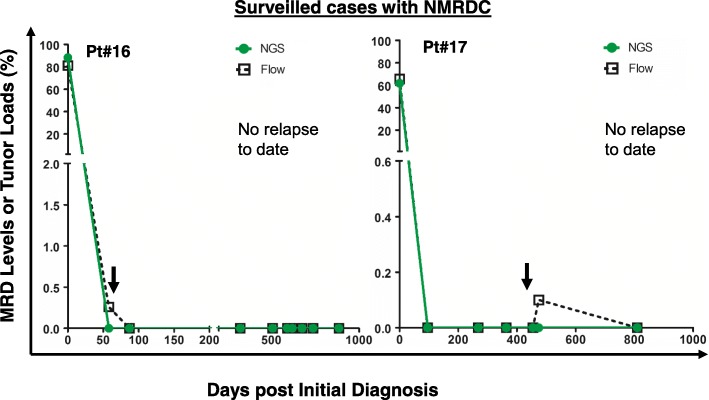
Representative surveilled B-ALL cases negative for MRD conversion (NMRDC). Dynamic changes in tumor load were measured by both the LIGV assay and MCF. Arrows indicate discordant MRD values where there was detectable MRD by MCF but negative NGS-MRD

Discordant results were observed in the remaining 25 follow-up samples. Among those specimens, 22 (18.03%) were MRD negative by MFC but positive by the NGS test, at very low levels ranging from 0.000174 to 0.30% with a median of 0.02242% (Fig. 3c and Additional file 7, the samples marked as red). MFC+/NGS- discordance was observed in the other three samples (Additional file 7, the samples marked as blue). This difference possibly represents false positivity by MFC, supported by the observation that these “MRD positive” cases have not been associated with any clinical relapses to date (Fig. 4).

The specificity of the assay was assessed by performing the MRD testing in patient-specific follow-up vs unrelated B-ALL samples. For this purpose, four patients who had both an identifiable leukemic-specific *IGH* clonotype as well as one diagnostic plus three follow-up samples available including relapsed specimens, were selected (Additional file 8). Besides four samples for each individual patient, additional 15 unrelated B-ALL and 5 normal PBMC control samples were assayed for the patient-specific tumor burdens. As shown in Additional file 8, while the leukemia-specific *IGH* clonotypes were detectable in diagnostic and/or other follow-up samples for each individual patient, no such clones were detected in all the unrelated B-ALL and normal PBMC samples (Additional file 8), demonstrating 100% specificity for the NGS assay as well as absence of experimental or bioinformatics contamination.

### Post remission MRD surveillance by NGS but not MCF can detect early conversion to positive MRD (CPMRD) before clinical relapse

**Fig. 5:**
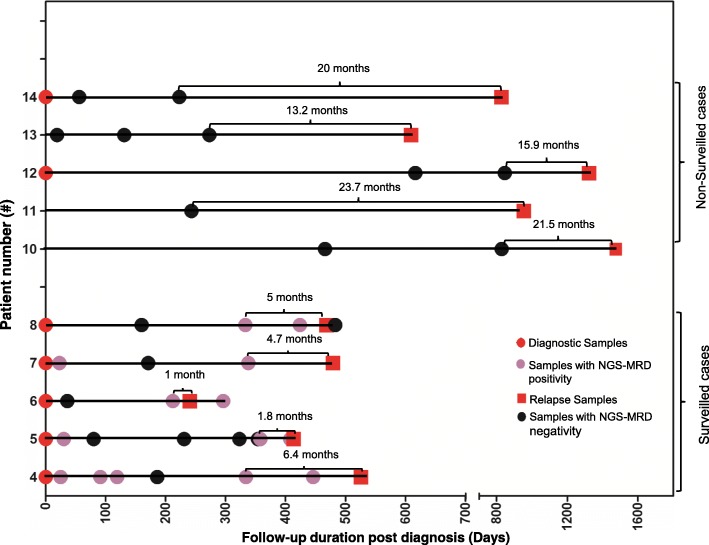
Chronology of specimen collections in the surveilled and non-surveilled relapsed B-ALL cases. Each horizontal line represents one patient with B-ALL relapse. The mean time interval between the most recent follow-up sample and overt relapse (marked) in the non-surveilled cases is significantly longer compared to that between conversion to positive MRD (CPMRD) and overt relapse (marked) in surveilled cases (20 months vs 4.7 months, *p* < 0.0001). See Fig. 6 for treatment information

**Fig. 6:**
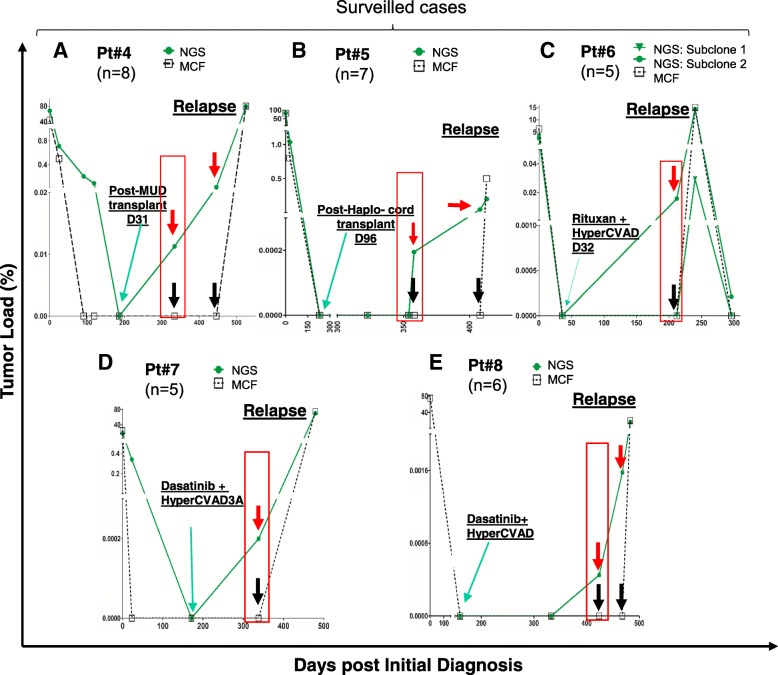
Early detection of CPMRD by NGS in surveilled patients. Serial measurements of tumor loads from five closely surveilled B-ALL cases were quantified by NGS and MCF assays. Two of the five patients (**a**, Pt #4 **b**, Pt #5) were post-transplant, and the other three patients (**c**, **d**, **e**)  were status-post hyperCVAD chemotherapy plus Rituxan or Dasatinib. In all patients (**a**, **b**, **c**, **d**, **e**), MRD was not detected by either NGS or MCF after treatment (green arrow). Conversion to positive MRD (CPMRD) demonstrated by NGS was highlighted by red boxes in each patient. Discordant MRD values where there is positive NGS MRD but no detectable MRD by MCF were highlighted by red arrows (NGS-based MRD levels) and black arrows (MCF-based MRD levels). MUD, matched unrelated donor, Pt#, patient number, n, number of samples collected from each individual patient

We hypothesized that this NGS test, with its superior sensitivity as compared to the MCF, can detect conversion to positive MRD (CPMRD) from undetectable MRD with MRD surveillance at a much earlier stage of relapse. To test this hypothesis and also to determine whether CPMRD may be useful to predict eventual relapse, 10 patients were selected for this comparison study according to the following four criteria: (1) positive identification of leukemia-specific clonotypes; (2) achievement of complete molecular remission defined as undetectable MRD by NGS before relapse; (3) availability of sufficient amount (minimum 500 ng) of DNA from post CR, pre-relapse specimens; and (4) symptomatic clinical relapse after achieving CR as described above. All patients were initially treated by conventional chemotherapy, with patients #4, #5, and #14 further undergoing stem cell transplantation. These patients were classified into two groups, surveilled (*n* = 5) and non-surveilled (*n* = 5), according to whether any post-treatment bone marrow specimens for ILGV assay are available within 6–7 months prior to clinical relapse (Fig. 5). The leukemic contents at different time points are shown in Fig. 6.

All surveilled patients achieved MRD negativity based on MCF and NGS post-chemotherapy. In patient #4, at day 186 post-initial diagnosis (day 37 post-matched unrelated donor (MUD) stem cell transplantation), the patient achieved molecular MRD response (Figs. 5 and 6a). The tumor burden then started to increase slowly, with the MRD levels increasing two folds (0.0113% vs 0.0238%) during the 4-month interval (day 334 to day 446). Then the tumor appeared to grow more rapidly, with a 3336.5-fold increase in tumor content in a span of 80 days (Fig. 6a). In patient #5, CPMRD was detected by NGS at day 358. From days 358 to 408, patient #5’s tumor burden increased 304 folds from 0.000195 to 0.0593%, with an average daily increase of 6.1 folds, followed by clinical relapse on day 413 (Fig. 6b). In patients #6–8, CPMRDs were also captured early by NGS. Patients #6, #7, and #8 achieved molecular remission on days 36, 171, and 160 post initial diagnosis and were converted to NGS-MRD positivity on days 212, 338, and 333 post initial diagnosis, respectively (Figs. 5 and 6c–e), each having 324-, 1063.8-, and 6.2-fold increase in leukemic burden before hematologic relapse. All patients with CPMRD eventually relapsed, and the median interval between CPMRD to clinical relapse in the surveilled cases is 4.7 months (1 to 6.4 months, Fig. 5). In all these patients, the emergence of relapsed tumor clones were detected earlier by NGS compared to MCF. In fact, MCF provided false negative results in all measurable time points before clinical relapse.

On the other hand, among the non-surveilled cases (patients # 10–14), all the patients had relapses despite the lack of demonstrable CPMRD. In this group of patients, CPMRD most likely has escaped detection in these patients because of the lack of sampling in the immediate months before clinical relapse. In line with this explanation, the median interval between the most recent post-treatment sample and clinical relapse in this group of patients was 20 months (13.2 to 23.7 months, Fig. 5), which was significantly longer compared to the interval between the detection of CPMRD and overt relapse in the surveilled patients (20 months vs 4.7 months, *p* = 0.0001) (Fig. 5). These findings suggest the utility of more frequent MRD surveillance based on *IGH* deep sequencing in a post-remission clinical setting for the detection of early relapse.

In addition to the surveilled cases with CPMRD, there are also some cases without CPMRD during the observation period (*n* = 10). For example, as shown in Fig. 4, patients #16 and #17 became MRD negative by both methods at day 87 and 96 post initial diagnosis and have remained MRD negative since then to date (days 885 and 810 post initial diagnosis, respectively). Their median intervals for serial bone marrow sampling were 2 and 3.1 months, respectively. All patients have not had any recurring disease to date.

Our findings support an association between CPMRD detection and eventual clinical relapse. Overall, these data demonstrated that post-remission MRD surveillance with NGS is superior to conventional MFC and offers a highly sensitive method to capture positive MRD conversion at earlier time points. This approach potentially expands the time window for preemptive therapies.

### Superior predictive power of post-remission CPMRD detected by NGS-based MRD surveillance for clinical relapse

**Fig. 7:**
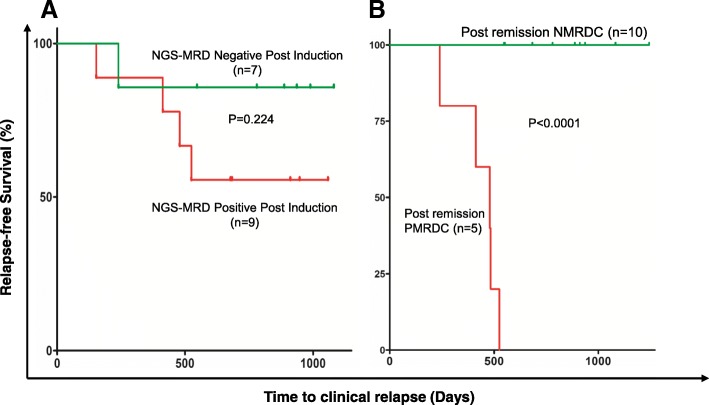
Prediction of clinical relapse based on NGS-detected MRD status post-induction and post-remission CPMRD. **a** Kaplan-Meier relapse-free survival curves for patients with negative or positive NGS-MRD post induction (between days 30 and 90). **b** Kaplan-Meier relapse-free survival curves for patients with post remission conversion to positive MRD (CPMRD) or without conversion (NMRDC)

As stated earlier, previous large studies have unambiguously demonstrated predictive power of MRD levels at the end of induction (day 29 or week 5–16) for B-ALL relapse, primarily based on qPCR or flow cytometry [2, 3, 6, 24–27]. However, using those methods, some of B-ALL patients with positive MRD at the end of induction were found to have no relapse, and 30–40% of adult B-ALL with negative MRD at the end of induction or after achieving CR clinically relapsed [8, 9, 28]. Along the same line, we would like to determine if detection of MRD by NGS at the end of induction has similar prognostic significance in our cohort. To test this, 16 evaluable B-ALL patients were classified into two groups, NGS-MRD negative (*n* = 7) and positive (*n* = 9), according to whether the patients achieved NGS-based MRD negativity at the end of induction (within initial 3–4 months of treatment). Analysis of relapse-free survival by Kaplan-Meier method showed that the patients with NGS-MRD positivity had a relatively but not statistically significant shorter time to relapse than the patients who achieved NGS-MRD negativity post-induction (*p* = 0.224 by the log-rank test, Fig. 7a). The relative risk of relapse in patients with NGS-MRD positivity was 2.84 times higher than in those with NGS-MRD negativity (95% CI, 0.4886 to 16.55). However, this difference was not significant (*p* = 0.224). It is conceivable that these results may be confounded by the small cohort size, but it is apparent from this limited study that about half of the patients with positive MRD by NGS did not relapse within the observation period.

We next investigated if CPMRD identified by NGS-based post-remission MRD surveillance could serve as a better marker to accurately predict clinical relapse. Eligible patients were classified into NMRDC or CPMRD groups according to the results of NGS-based serial MRD quantitative surveillance, and time to clinical relapse was calculated. NMRDC and CPMRD groups included 10 and 5 patients, respectively, and the time to clinical relapse (days) was defined as the duration from the day of diagnosis to the day of relapse or to the last available date if relapse has not occurred to Jan. 20, 2018. Kaplan-Meier analysis of relapse-free survival showed that the patients with CPMRD after CR1 had a significantly shorter time to relapse than the patients with NMRDC (median time from diagnosis to relapse: 479 days vs not reached, *p* < 0.0001 by the log-rank test, Fig. 7b). CPMRD was associated with an impending clinical relapse in the near future (hazard ratio, 111.2, 95% confidence interval: 12.92 to 996.5) regardless of modality of treatment (chemotherapy or bone marrow transplantation), and none of the patients without CPMRD relapsed. CPMRD in our limited patient cohort had a clinical sensitivity of 100% and a clinical specificity of 100% with a positive predictive value (PPV) of 1 and a negative predictive value (NPV) of 1 for clinical relapse (*p* = 0.0006). These results suggest that CPMRD in post-remission MRD surveillance setting may serve as a reliable marker for clinical relapse prediction.

## Discussion

We have evaluated and validated a simple and sensitive NGS-based MRD detection method for patients with B-ALL, which has an excellent potential to be adopted in clinical settings. Compared to the current NGS platform for B-ALL MRD qualification, which is proprietary and requires specimen send-out to a central commercial laboratory, the LIGV-Miseq method is simpler and can be easily implemented in a routine clinical molecular laboratory. We described a pilot proof-of-concept study to document its clinical utility in routine MRD surveillance and early detection of CPMRD in post-chemotherapy and post bone marrow transplantation settings. Detection of CPMRD reliably predicts impending clinical relapse, potentially expanding the time window for more effective preemptive therapies against impending relapsed B-ALL.

Several groups have explored the potential of NGS-based MRD detection assays and showed exceptional sensitivity and accuracy [10, 12–14, 17, 21, 29, 30]. However, platforms like LymphoSIGHT (Sequenta) were hardly applicable in a conventional clinical laboratory. In this study, evaluation of key analytical performance characteristics demonstrates that the LIGV-Miseq platform is an excellent alternative that is applicable in > 90% of B-ALL cases. The current method uses single rather than several rounds of multiplex PCR (as in LymphoSIGHT method) for sequencing library preparation, minimizing labor time and the potential for human error and contamination. The sensitivity of our method reaches 10^− 6^ if adequate DNA input is available. This makes this approach superior to MCF [21] as evidenced by the observation that NGS detected MRD in 22 MCF-based MRD-negative samples (Fig. 3c), and it may be better or at least comparable to alternative, much more demanding, assays [10, 12–14, 17, 29, 30]. Our NGS method also demonstrates excellent intra-run and inter-run reproducibility near the established limit of detection. High concordance with MCF results is observed across a broad range of tumor load, demonstrating high accuracy of the LIGV-Miseq MRD testing platform. These findings support our NGS platform as a simple, sensitive, and reliable assay for MRD monitoring in B-ALL patients, and a practical option for a routine clinical laboratory.

The highly quantitative results generated with this assay from our retrospective cohort of B-ALL patients support the clinical utility of NGS-based post-remission MRD surveillance. As far as we know, the current study is the first that evaluates the clinical significance of NGS-based MRD surveillance during the period of complete remission by retrospectively analyzing serial post-treatment samples. There appears to be a strong correlation between CPMRD and clinical relapse, suggesting that CPMRD may signify impending relapse. Several previous investigations demonstrated the clinical potential and prognostic value of flow cytometry or qPCR-based MRD quantification in post-remission setting, producing the lead times from clinical relapse of 3.6 and 4.1 months, respectively [8, 28]. However, the accuracy of prediction of relapse for these MRD determinations was less than 100%. Some of the patients with MRD positivity by a flow cytometry method never underwent hematologic relapse, and a fraction of the patients with MRD negativity relapsed clinically (up to 30–40% for some studies) [7, 8, 28]. The occurrence of relapse with prior MRD negativity may be attributable to the limited sensitivity of the MFC or qPCR-based assays as suggested by our data (Figs. 3d and 6), or as seen in our non-surveilled cases, to insufficient surveillance since MRD surveillance is not routinely performed in many post remission settings. False positive readouts, i.e., positive MRD but no eventual relapse, might suggest stable or even decreasing MRD, which again could not have been detected unless periodic MRD measurements are made. As suggested by our results, it is possible that the determining predictive factor for relapse is not the presence or absence of MRD per se, but a rising trend of MRD, which, in the cases of prior molecular remission, is equivalent to CPMRD. Our results support the potential clinical relevance of NGS-based MRD surveillance involving periodic MRD measurements to detect CPMRD. Whether a continuous increase in MRD in cases without molecular remission has the same predictive value as CPMRD awaits further investigation.

The dynamic quantitative results generated by this assay during post treatment MRD surveillance may serve as a highly useful biomarker in patients with B-ALL for early detection of relapse disease. Early relapse detection widens the window for preemptive therapy against overt relapse. Implementation of preemptive therapies such as CAR T immunotherapy and allogenic stem cell transplantation has been associated with significant responses and prolonged survival with fewer side effects in relapsed patients with lower leukemia burdens [22, 31, 32]. A number of studies have shown that the efficacy of preemptive therapies, such as withdrawal of immunosuppression or infusion of donor lymphocyte, is closely correlated with low leukemia burdens [32, 33]. A long-term follow-up clinical trial showed that relapsed B-ALL patients with a low disease burden, who were treated with CD19 CAR T Therapy, had longer long-term survival and a lower incidence of the cytokine release syndrome and neurotoxic events than did patients with a higher tumor load [22], supporting critical role of early detection of CPMRD in effectively managing relapsed patients with preemptive therapy like CAR T immunotherapy. MRD surveillance should be taken into account in modern B-ALL management protocol and to guide personalized therapy decisions. The superb precision and analytic sensitivity of this test is particularly suited for generating highly quantitative results for determination of molecular remission and pre-clinical molecular relapse.

Since the size of our cohort is limited, the clinical utility of MRD surveillance needs to be further verified in a larger prospective clinical study. Confounding factors may limit the clinical utility/performance of the LIGV-Miseq platform. First, leukemia-specific clonotypes were not identified in a small subset of patients (2 of 32, ~ 6.25%). The reason is likely due to V gene deletion or incomplete DJ rearrangement during evolution of leukemia clones [33, 34]. To address this problem, integration of clonal *IgH* DJ rearrangement detection may be one of the options, potentially increasing the applicability of the test to more than 96% of B-ALL cases. The second limitation factor is availability of sufficient DNA material. Although most of the bone marrow samples produce substantial amount of DNA, those collected from B-ALL with severe BM fibrosis may be difficult to yield enough DNA for NGS-based MRD monitoring [35].

## Conclusion

In summary, we have described and characterized a simple, reliable, and highly sensitive NGS platform that can be easily implemented in a routine molecular laboratory for MRD testing of B-ALL. Moreover, using this platform, we demonstrate the clinical utility of a dynamic NGS-based post-remission MRD surveillance method for better risk stratification and earlier preemptive therapies against impending relapse, thus potentially improving outcome for B-ALL patients. Besides B-ALL, the current NGS-based MRD testing platform should be readily applicable to MRD monitoring in other B-cell neoplasms, to assess response to therapies with innovative drugs and to design tailored protocols involving minimal disease detection in patients with B-cell lymphoproliferative disorders.

## Additional files

Additional file 1: Overview of MRD measurement in B-ALL. (1) Genomic DNAs were extracted from evaluable pre-treatment and post-treatment B-ALL samples; (2) VDJ rearrangements were amplified with indicated indexed primer sets (FR3, FR1, or Leader with J_H_) in a single multiplex PCR reaction to capture all immunoglobulin heavy-chain VDJ rearrangements. For the forward primers, we recommend using FR3 first. If no clonality were detected, FR1 or Leader may be used. The same sets of primer pairs were used for the initial diagnostic and corresponding follow-up specimens; (3) Resulting libraries were purified and quantified, followed by Miseq sequencing; (4) A customized algorithm was used to analyze NGS data to identify clonal *IGH* VDJ rearrangements for diagnostic specimens and to generate MRD values for post-treatment specimens. (DOCX 129 kb)

Additional file 2: Observed and expected MRD levels after serial dilution in representative B-ALL samples. (XLSX 15 kb)

Additional file 3: Intra-run reproducibility of LIGV-Miseq assay in MRD detection. (DOCX 13 kb)

Additional file 4: Inter-run reproducibility of LIGV-Miseq assay in MRD detection. (DOCX 12 kb)

Additional file 5: Patient characteristic. (DOCX 12 kb)

Additional file 6: Correlation between two NGS-based tumor load measurement methods. *X*-axis represents tumor load calculated by using B-cell fraction size in mononuclear cell populations measured by flow cytometry; *Y*-axis, tumor load based on the spike-in method. More details for the derivation of tumor load values based on these two methods are described in Material & Methods section. r, correlation coefficient, n, number of samples. (DOCX 63 kb)

Additional file 7: Tumor load measured by NGS vs MCF assays for all evaluable diagnostic and follow-up B-ALL samples. (XLSX 14 kb)

Additional file 8: Tumor load or MRD levels measured by NGS and MCF in patient related and unrelated samples. (XLSX 14 kb)

## Abbreviations

ASO-PCR: Allele-specific oligonucleotide PCR
B-ALL: B acute lymphoblastic leukemia/lymphoma
CDR3: Complementary determining region 3
CPMRD: Conversion to positive MRD
CR: Complete remission
IGH: Immunoglobulin heavy chain
LIGV-Miseq: LymphoTrack® IGHV-based deep sequencing assay (Miseq platform)
MCF: Multi-color flow cytometry
MRD: Minimal (measurable) residual disease
MUD: Matched unrelated donor
NGS: Next-generation sequencing
NMRDC: Negative MRD conversion
NPV: Negative predictive value
PPV: Positive predictive value

## References

[1] Ciudad J, San Miguel JF, Lopez-Berges MC, Vidriales B, Valverde B, Ocqueteau M, Mateos G, Caballero MD, Hernandez J, Moro MJ (1998). Prognostic value of immunophenotypic detection of minimal residual disease in acute lymphoblastic leukemia. J Clin Oncol.

[2] Cave H, van der Werff ten Bosch J, Suciu S, Guidal C, Waterkeyn C, Otten J, Bakkus M, Thielemans K, Grandchamp B, Vilmer E (1998). Clinical significance of minimal residual disease in childhood acute lymphoblastic leukemia. European Organization for Research and Treatment of Cancer—Childhood Leukemia Cooperative Group. N Engl J Med.

[3] Borowitz MJ, Devidas M, Hunger SP, Bowman WP, Carroll AJ, Carroll WL, Linda S, Martin PL, Pullen DJ, Viswanatha D (2008). Clinical significance of minimal residual disease in childhood acute lymphoblastic leukemia and its relationship to other prognostic factors: a children’s oncology group study. Blood.

[4] Conter V, Bartram CR, Valsecchi MG, Schrauder A, Panzer-Grumayer R, Moricke A, Arico M, Zimmermann M, Mann G, De Rossi G (2010). Molecular response to treatment redefines all prognostic factors in children and adolescents with B-cell precursor acute lymphoblastic leukemia: results in 3184 patients of the AIEOP-BFM ALL 2000 study. Blood.

[5] Zugmaier G, Gokbuget N, Klinger M, Viardot A, Stelljes M, Neumann S, Horst HA, Marks R, Faul C, Diedrich H (2015). Long-term survival and T-cell kinetics in relapsed/refractory ALL patients who achieved MRD response after blinatumomab treatment. Blood.

[6] van Dongen JJ, van der Velden VH, Bruggemann M, Orfao A (2015). Minimal residual disease diagnostics in acute lymphoblastic leukemia: need for sensitive, fast, and standardized technologies. Blood.

[7] Dworzak MN, Froschl G, Printz D, Mann G, Potschger U, Muhlegger N, Fritsch G, Gadner H (2002). Prognostic significance and modalities of flow cytometric minimal residual disease detection in childhood acute lymphoblastic leukemia. Blood.

[8] Raff T, Gokbuget N, Luschen S, Reutzel R, Ritgen M, Irmer S, Bottcher S, Horst HA, Kneba M, Hoelzer D (2007). Molecular relapse in adult standard-risk ALL patients detected by prospective MRD monitoring during and after maintenance treatment: data from the GMALL 06/99 and 07/03 trials. Blood.

[9] Bruggemann M, Raff T, Flohr T, Gokbuget N, Nakao M, Droese J, Luschen S, Pott C, Ritgen M, Scheuring U (2006). Clinical significance of minimal residual disease quantification in adult patients with standard-risk acute lymphoblastic leukemia. Blood.

[10] Faham M, Zheng J, Moorhead M, Carlton VE, Stow P, Coustan-Smith E, Pui CH, Campana D (2012). Deep-sequencing approach for minimal residual disease detection in acute lymphoblastic leukemia. Blood.

[11] Pulsipher MA, Carlson C, Langholz B, Wall DA, Schultz KR, Bunin N, Kirsch I, Gastier-Foster JM, Borowitz M, Desmarais C (2015). IgH-V(D)J NGS-MRD measurement pre- and early post-allotransplant defines very low- and very high-risk ALL patients. Blood.

[12] Boyd SD, Marshall EL, Merker JD, Maniar JM, Zhang LN, Sahaf B, Jones CD, Simen BB, Hanczaruk B, Nguyen KD (2009). Measurement and clinical monitoring of human lymphocyte clonality by massively parallel VDJ pyrosequencing. Sci Transl Med.

[13] Logan AC, Vashi N, Faham M, Carlton V, Kong K, Buno I, Zheng J, Moorhead M, Klinger M, Zhang B (2014). Immunoglobulin and T cell receptor gene high-throughput sequencing quantifies minimal residual disease in acute lymphoblastic leukemia and predicts post-transplantation relapse and survival. Biol Blood Marrow Transplant.

[14] Kotrova M, Muzikova K, Mejstrikova E, Novakova M, Bakardjieva-Mihaylova V, Fiser K, Stuchly J, Giraud M, Salson M, Pott C (2015). The predictive strength of next-generation sequencing MRD detection for relapse compared with current methods in childhood ALL. Blood.

[15] Wu J, Jia S, Wang C, Zhang W, Liu S, Zeng X, Mai H, Yuan X, Du Y, Wang X (2016). Minimal residual disease detection and evolved IGH clones analysis in acute B lymphoblastic leukemia using IGH deep sequencing. Front Immunol.

[16] Wu D, Emerson RO, Sherwood A, Loh ML, Angiolillo A, Howie B, Vogt J, Rieder M, Kirsch I, Carlson C (2014). Detection of minimal residual disease in B lymphoblastic leukemia by high-throughput sequencing of IGH. Clin Cancer Res.

[17] Ladetto M, Bruggemann M, Monitillo L, Ferrero S, Pepin F, Drandi D, Barbero D, Palumbo A, Passera R, Boccadoro M (2014). Next-generation sequencing and real-time quantitative PCR for minimal residual disease detection in B-cell disorders. Leukemia.

[18] Meek K (1990). Analysis of junctional diversity during B lymphocyte development. Science.

[19] Komori T, Okada A, Stewart V, Alt FW (1993). Lack of N regions in antigen receptor variable region genes of TdT-deficient lymphocytes. Science.

[20] Gilfillan S, Dierich A, Lemeur M, Benoist C, Mathis D (1993). Mice lacking TdT: mature animals with an immature lymphocyte repertoire. Science.

[21] Wood B, Wu D, Crossley B, Dai Y, Williamson D, Gawad C, Borowitz MJ, Devidas M, Maloney KW, Larsen E (2018). Measurable residual disease detection by high-throughput sequencing improves risk stratification for pediatric B-ALL. Blood.

[22] Park JH, Riviere I, Gonen M, Wang X, Senechal B, Curran KJ, Sauter C, Wang Y, Santomasso B, Mead E (2018). Long-term follow-up of CD19 CAR therapy in acute lymphoblastic leukemia. N Engl J Med.

[23] Sala Torra O, Othus M, Williamson DW, Wood B, Kirsch I, Robins H, Beppu L, O'Donnell MR, Forman SJ, Appelbaum FR, Radich JP (2017). Next-generation sequencing in adult B cell acute lymphoblastic leukemia patients. Biol Blood Marrow Transplant.

[24] Pieters R, de Groot-Kruseman H, Van der Velden V, Fiocco M, van den Berg H, de Bont E, Egeler RM, Hoogerbrugge P, Kaspers G, Van der Schoot E (2016). Successful therapy reduction and intensification for childhood acute lymphoblastic leukemia based on minimal residual disease monitoring: study ALL10 from the Dutch childhood oncology group. J Clin Oncol.

[25] Paganin M, Fabbri G, Conter V, Barisone E, Polato K, Cazzaniga G, Giraldi E, Fagioli F, Arico M, Valsecchi MG, Basso G (2014). Postinduction minimal residual disease monitoring by polymerase chain reaction in children with acute lymphoblastic leukemia. J Clin Oncol.

[26] Bassan R, Spinelli O, Oldani E, Intermesoli T, Tosi M, Peruta B, Rossi G, Borlenghi E, Pogliani EM, Terruzzi E (2009). Improved risk classification for risk-specific therapy based on the molecular study of minimal residual disease (MRD) in adult acute lymphoblastic leukemia (ALL). Blood.

[27] Bader P, Kreyenberg H, Henze GH, Eckert C, Reising M, Willasch A, Barth A, Borkhardt A, Peters C, Handgretinger R (2009). Prognostic value of minimal residual disease quantification before allogeneic stem-cell transplantation in relapsed childhood acute lymphoblastic leukemia: the ALL-REZ BFM study group. J Clin Oncol.

[28] Pemmaraju N, Kantarjian H, Jorgensen JL, Jabbour E, Jain N, Thomas D, O'Brien S, Wang X, Huang X, Wang SA (2017). Significance of recurrence of minimal residual disease detected by multi-parameter flow cytometry in patients with acute lymphoblastic leukemia in morphological remission. Am J Hematol.

[29] Salson M, Giraud M, Caillault A, Grardel N, Duployez N, Ferret Y, Duez M, Herbert R, Rocher T, Sebda S (2016). High-throughput sequencing in acute lymphoblastic leukemia: follow-up of minimal residual disease and emergence of new clones. Leuk Res.

[30] Logan AC, Zhang B, Narasimhan B, Carlton V, Zheng J, Moorhead M, Krampf MR, Jones CD, Waqar AN, Faham M (2013). Minimal residual disease quantification using consensus primers and high-throughput IGH sequencing predicts post-transplant relapse in chronic lymphocytic leukemia. Leukemia.

[31] Liu J, Zhong JF, Zhang X, Zhang C (2017). Allogeneic CD19-CAR-T cell infusion after allogeneic hematopoietic stem cell transplantation in B cell malignancies. J Hematol Oncol.

[32] Nagafuji K, Miyamoto T, Eto T, Kamimura T, Taniguchi S, Okamura T, Ohtsuka E, Yoshida T, Higuchi M, Yoshimoto G (2013). Monitoring of minimal residual disease (MRD) is useful to predict prognosis of adult patients with Ph-negative ALL: results of a prospective study (ALL MRD2002 study). J Hematol Oncol.

[33] Buckley SA, Appelbaum FR, Walter RB (2013). Prognostic and therapeutic implications of minimal residual disease at the time of transplantation in acute leukemia. Bone Marrow Transplant.

[34] Buccisano F, Maurillo L, Del Principe MI, Del Poeta G, Sconocchia G, Lo-Coco F, Arcese W, Amadori S, Venditti A (2012). Prognostic and therapeutic implications of minimal residual disease detection in acute myeloid leukemia. Blood.

[35] Noren-Nystrom U, Roos G, Bergh A, Botling J, Lonnerholm G, Porwit A, Heyman M, Forestier E (2008). Bone marrow fibrosis in childhood acute lymphoblastic leukemia correlates to biological factors, treatment response and outcome. Leukemia..

