# Supplementary material for: Simple deep sequencing-based post-remission MRD surveillance predicts clinical relapse in B-ALL
Source: J Hematol Oncol. 2018 Aug 22;11:105. doi: 10.1186/s13045-018-0652-y (PMC6103872; doi:10.1186/s13045-018-0652-y)
Supplement: Supplementary file 1 — Overview of MRD measurement in B-ALL. (1) Genomic DNAs were extracted from evaluable pre-treatment and post-treatment B-ALL samples; (2) VDJ rearrangements were amplified with indicated indexed primer sets (FR3, FR1, or Leader with JH) in a single multiplex PCR reaction to capture all immunoglobulin heavy-chain VDJ rearrangements. For the forward primers, we recommend using FR3 first. If no clonality were detected, FR1 or Leader may be used. The same sets of primer pairs were used for the initial diagnostic and corresponding follow-up specimens; (3) Resulting libraries were purified and quantified, followed by Miseq sequencing; (4) A customized algorithm was used to analyze NGS data to identify clonal IGH VDJ rearrangements for diagnostic specimens and to generate MRD values for post-treatment specimens. (DOCX 129 kb) [file 13045_2018_652_MOESM1_ESM.docx]

**Additional file 1:** **Overview of MRD measurement in B-ALL**. (1) Genomic DNAs were extracted from evaluable pre-treatment and post-treatment B-ALL samples; (2) VDJ rearrangements were amplified with indicated indexed primer sets (FR3, FR1, or Leader with J_H_) in a single multiplex PCR reaction to capture all immunoglobulin heavy chain VDJ rearrangements. For the forward primers, we recommend using FR3 first. If no clonality were detected, FR1 or Leader may be used. The same sets of primer pairs were used for the initial diagnostic and corresponding follow-up specimens; (3) Resulting libraries were purified and quantified, followed by Miseq sequencing; (4) A customized algorithm was used to analyze NGS data to identify clonal *IGH* VDJ rearrangements for diagnostic specimens and to generate MRD values for post-treatment specimens.

**(1) Genomic DNA Extraction**


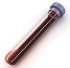

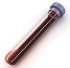


Diagnostic specimens

(Bone marrow or PBL)

Post-treatment specimen(s)

(Bone marrow or PBL)

**(2) Barcoded LymphoTrack Multiplex PCR**

Rearranged

*IGH*

**L**

**V**

**L**

**V**

**D**

**J**

**L**

**V**

**V_H_ FR1**

**Forward primers**

**J_H_ primers**

**D_H_**

**J_H_**

**L_H_**

**V_H_**

**(3) Miseq Deep Sequencing**


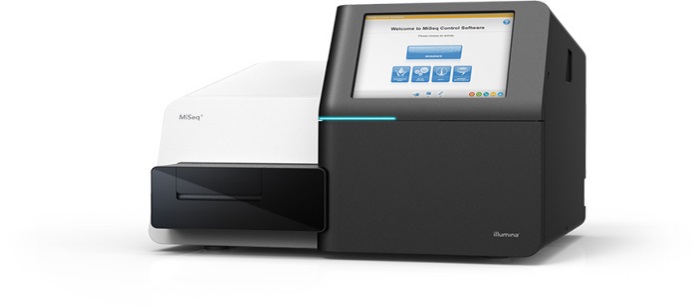


**(4) Data analysis and Reporting**


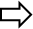


**V_H_ FR3**

**V_H_L**

**Reverse primers**

**LIGV-Miseq MRD detection method**
