# Supplementary material for: Simple deep sequencing-based post-remission MRD surveillance predicts clinical relapse in B-ALL
Source: J Hematol Oncol. 2018 Aug 22;11:105. doi: 10.1186/s13045-018-0652-y (PMC6103872; doi:10.1186/s13045-018-0652-y)
Supplement: Supplementary file 3 — Intra-run reproducibility of LIGV-Miseq assay in MRD detection. (DOCX 13 kb) [file 13045_2018_652_MOESM3_ESM.docx]

|  | | | | | | |
| --- | --- | --- | --- | --- | --- | --- |
| **B-ALL Sample ID** | **Barcoded Intra-Run Samples** | | | **Mean** | **SD** |  |
|  | **#1** | **#2** | **#3** |  |  |  |
|  | **B-ALL MRD (% of total cells)** | | | | |  |
| 3623 | 0.00071 | 0.00081 | 0.00195 | 0.00116 | 0.00069 |  |
| 3501 | 0.000096 | 0.00037 | 0.00049 | 0.00032 | 0.00020 |  |
| 3064 | 0.00079 | 0.00042 | 0.00022 | 0.00048 | 0.00029 |  |
|  | **Leukemia-Specific Reads** | | | | |  |
| 3623 | 43 | 44 | 214 | 100 | 98 |  |
| 3501 | 7 | 19 | 22 | 16 | 8 |  |
| 3064 | 11 | 29 | 7 | 16 | 12 |  |
|  | **Total Reads** | | | | |  |
| 3623 | 486759 | 432556 | 878992 | 599436 | 243615 |  |
| 3501 | 584790 | 415113 | 358524 | 452809 | 117749 |  |
| 3064 | 111183 | 558626 | 254226 | 308012 | 228519 |  |
| B-ALL, B-lineage acute lymphoblastic leukemia/lymphoma | | | |  |  |  |
| SD, standard deviation, MRD, minimal residual disease | | | |  |  |  |

**Additional file 3: Intra-run reproducibility of LIGV– Miseq assay in MRD detection**
