# Supplementary material for: Simple deep sequencing-based post-remission MRD surveillance predicts clinical relapse in B-ALL
Source: J Hematol Oncol. 2018 Aug 22;11:105. doi: 10.1186/s13045-018-0652-y (PMC6103872; doi:10.1186/s13045-018-0652-y)
Supplement: Supplementary file 4 — Inter-run reproducibility of LIGV-Miseq assay in MRD detection. (DOCX 12 kb) [file 13045_2018_652_MOESM4_ESM.docx]

**Additional file 4**: **Inter-run reproducibility of LIGV– Miseq assay in MRD detection**

|  | | | | | | | |
| --- | --- | --- | --- | --- | --- | --- | --- |
| **B-ALL Sample ID** | **Number of Run** | | | | | **Mean** | **SD** |
|  | **1** | **2** | **3** | **4** | **5** |  |  |
| 3623 | **B-ALL MRD (% of total cells)** | | | | | | |
|  | 0.00094 | 0.00110 | 0.00049 | 0.00072 | 0.00115 | 0.00088 | 0.000275 |
|  | **Leukemia-Specific Reads** | | | | | | |
|  | 68 | 328 | 17 | 117 | 270 | 160 | 133 |
|  | **Total Reads** | | | | | | |
|  | 581533 | 2377744 | 277828 | 1301532 | 1878579 | 1283443 | 874064 |
| B-ALL, B-lineage acute lymphoblastic leukemia/lymphoma | | | | |  |  |  |
| SD, standard deviation, MRD, minimal residual disease | | | | |  |  |  |
