# Supplementary material for: Simple deep sequencing-based post-remission MRD surveillance predicts clinical relapse in B-ALL
Source: J Hematol Oncol. 2018 Aug 22;11:105. doi: 10.1186/s13045-018-0652-y (PMC6103872; doi:10.1186/s13045-018-0652-y)
Supplement: Supplementary file 5 — Patient characteristic. (DOCX 12 kb) [file 13045_2018_652_MOESM5_ESM.docx]

**Additional file 5: Patient Characteristic**

|  | |
| --- | --- |
| **VARIABLES** | **NUMBER (%)** |
| **Total Patient** | 30(100) |
| **Age, median (range, Yr)** | 47(2-82) |
|  |  |
| **Genetic Alteration** |  |
| Normal Karyotype | 5(16.7) |
| hyperdiploidy | 1(3.33) |
| *KMT2A* rearranged | 3 (10.0) |
| *BCR-ABL1* fused gene | 12 (40.0) |
| *Others* | 9 (30) |
|  |  |
| **Treatment** |  |
| Chemotherapy | 30 (100) |
| SCT transplant | 5 (16.7) |
| Radiotherapy | 1(3.3) |
| Combined | 10 (33.3) |
|  |  |
| **Clinical Outcome** |  |
| CR | 11(36.7%) |
| Relapse | 15 (50.0%) |
| Other | 4 (13.3%) |
| SCT, stem cell transplantation | |
| CR, complete remission |  |
