# Supplementary material for: Simple deep sequencing-based post-remission MRD surveillance predicts clinical relapse in B-ALL
Source: J Hematol Oncol. 2018 Aug 22;11:105. doi: 10.1186/s13045-018-0652-y (PMC6103872; doi:10.1186/s13045-018-0652-y)
Supplement: Supplementary file 6 — Correlation between two NGS-based tumor load measurement methods. X-axis represents tumor load calculated by using B-cell fraction size in mononuclear cell populations measured by flow cytometry; Y-axis, tumor load based on the spike-in method. More details for the derivation of tumor load values based on these two methods are described in Material & Methods section. r, correlation coefficient, n, number of samples. (DOCX 63 kb) [file 13045_2018_652_MOESM6_ESM.docx]

**Additional file 6: Correlation between two NGS-based tumor load measurement methods.** X axis represents tumor load calculated by using B-cell fraction size in mononuclear cell populations measured by flow cytometry; Y axis, tumor load based on the spike-in method. More details for the derivation of tumor load values based on these two methods are described in Material & Methods section. r, correlation coefficient, n, number of samples.


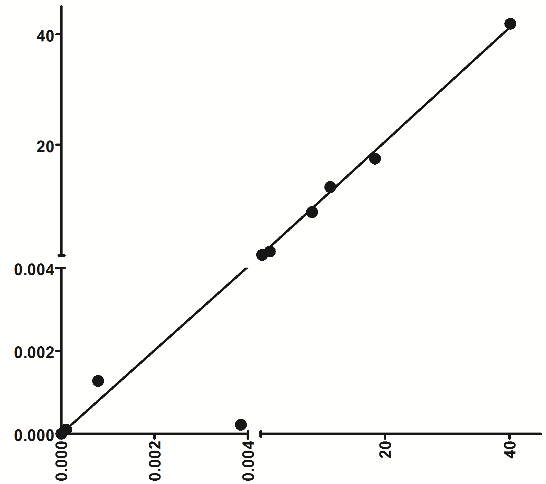


Tumor Load (%, based on B cell percentage

determined by Flow)

Tumor Load (%, determined by spike-in)

r = 0.99

P < 0.0001

n = 20
